# Wild-type cutoff for Apramycin against *Escherichia coli*

**DOI:** 10.1186/s12917-020-02522-0

**Published:** 2020-08-26

**Authors:** Yuqi Yang, Tianshi Xiao, Jiarui Li, Ping Cheng, Fulei Li, Hongxiao Yu, Ruimeng Liu, Ishfaq Muhammad, Xiuying Zhang

**Affiliations:** 1 Pharmacology Teaching and Research Department, School of Basic Medicine, Guizhou University of Traditional Chinese Medicine, Dongqing Road, University Town, Huaxi District, Guiyang, People’s Republic of China; 2 Heilongjiang Key Laboratory for Animal Disease Control and Pharmaceutical Development. Faculty of Basic Veterinary Science, College of Veterinary Medicine, Northeast Agricultural University, 600 Changjiang Road, Xiangfang District, Harbin, Heilongjiang 150030 People’s Republic of China

**Keywords:** Apramycin, *Escherichia coli*, Wild-type cutoff, Resistance, *Aac(3)-IV*

## Abstract

**Background:**

Apramycin is used exclusively for the treatment of *Escherichia coli* (*E.coli*) infections in swine around the world since the early 1980s. Recently, many research papers have demonstrated that apramycin has significant in vitro activity against multidrug-resistant *E.coli* isolated in hospitals. Therefore, ensuring the proper use of apramycin in veterinary clinics is of great significance of public health. The objectives of this study were to develop a wild-type cutoff for apramycin against *E.coli* using a statistical method recommended by Clinical and Laboratory Standards Institute (CLSI) and to investigate the prevalence of resistance genes that confer resistance to apramycin in *E. coli*.

**Results:**

Apramycin susceptibility testing of 1230 *E.coli* clinical isolates from swine were determinded by broth microdilution testing according to the CLSI document M07-A9. A total number of 310 *E.coli* strains from different minimum inhibitory concentration (MIC) subsets (0.5–256 μg/mL) were selected for the detection of resistance genes (*aac(3)-IV*; *npmA*; *apmA*) in *E. coli* by PCR. The percentage of *E. coli* isolates at each MIC (0.5, 1, 2, 4, 8, 16, 32, 64, 128, and 256 μg/mL) was 0.08, 0.08, 0.16, 2.93, 31.14, 38.86, 12.85, 2.03, 1.46, and 10.41%. The MIC_50_ and MIC_90_ were 16 and 64 μg/mL. All the 310 *E.coli* isolates were negative for *npmA* and *apmA* gene, and only the *aac(3)-IV* gene was detected in this study.

**Conclusions:**

The wild-type cutoff for apramycin against *E.coli* was defined as 32 μg/mL. The prevelance of *aac(3)-IV* gene mainly concentrated in these MIC subsets ‘MIC ≥ 64 μg/ mL’, which indicates that the wild-type cutoff established in our study is reliable. The wild-type cutoff offers interpretion criteria of apramycin susceptibility testing of *E.coli*.

## Background

*Escherichia coli* (*E.coli*) usually colonizes the animal gastrointestinal tract as a commensal bacterium, and only a small number of strains are pathogenic. Enterotoxigenic *E.coli* (ETEC) represents one of these pathotypes that cause a variety of enteric and extraintestinal diseases in humans and animals [1]. ETEC is spread by the fecal-oral route with food and water being the principal sources of infection [1]. In humans, ETEC is the main cause of bacterial diarrhea in adults and children in developing countries and is also a leading cause of traveler’s diarrhea [2]. In pigs, enteric diseases caused with ETEC may result in significant economic losses due to morbidity, mortality, cost for treatments, decreased weight gain, vaccinations, and feed supplements [3].

Apramycin (APR), an aminoglycoside antibiotic, has been used exclusively for the treatment of *E.coli* infections in swine, cattle, sheep, poultry, and rabbits around the world since the early 1980s and was approved for use in China in 1999 [4]. Recently, many research papers have demonstrated that apramycin has significant in vitro activity against multidrug-, carbapenem- and aminoglycoside-resistant *E.coli* isolated in hospitals. And its excellent breadth of activity renders apramycin a promising drug candidate for the treatment of systemic Gram-negative infections [5–11]. The first resistant *E. coli* strain was detectable in nature shortly after the application of APR [12]. It has been determined to date that two resistance genes confer resistance to APR in *E. coli*. One is AAC (3)-IV, which encodes an aminoglycoside 3-N-acetyltransferase type IV enzyme [13]. The other is NpmA, which was identified in a clinical *E. coli* strain and encodes a 16S rRNA m1A1408 methyltransferase [14]. Moreover, another APR resistance gene, apmA, was detected in bovine methicillin-resistant *Staphylococcus aureus* (MRSA) of sequence type 398 in 2011 and encodes for a protein of 274 amino acids [15]. APR resistance has been also detected in *E.coli* clinical isolates of hospitalized patients despite it has not been used in human medicine [16]. The horizontal transfer of the APR resistance gene *aac(3)-IV* results in the dissemination of APR-resistance *E. coli* isolates between animals and humans [17]. In addition, cross-resistance between APR and other aminoglycosides such as gentamicin (GEN) and tobramycin for the treatment of severe infections in humans has been well documented [18, 19]. Previous study reported that pigs may have been an important reservoir for GEN-resistance bacteria transfer to humans [20]. Considering the importance of GEN in human medicine, improper use of APR in animals contributing to increased resistance is of great concern.

Wild-type cutoff values (CO_WT_) are the useful tools available to laboratories performing susceptibility testing and to clinicians treating infections. In addition, the tools also provide alternative means for monitoring the emergence of drug resistance in any given bacterial species [21]. A statistical method was a more scientific method which has been adopted by the Clinical and Laboratory Standards Institute (CLSI) as a standard method for CO_WT_ establishment [22, 23]. The purposes of the present study were (i) to develop CO_WT_ of APR against *E.coli* using a statistical method recommended by CLSI and (ii) to investigate the prevalence of genes that confer resistance to APR in *E. coli*.

## Results

### Antibacterial susceptibility testing

**Fig. 1:**
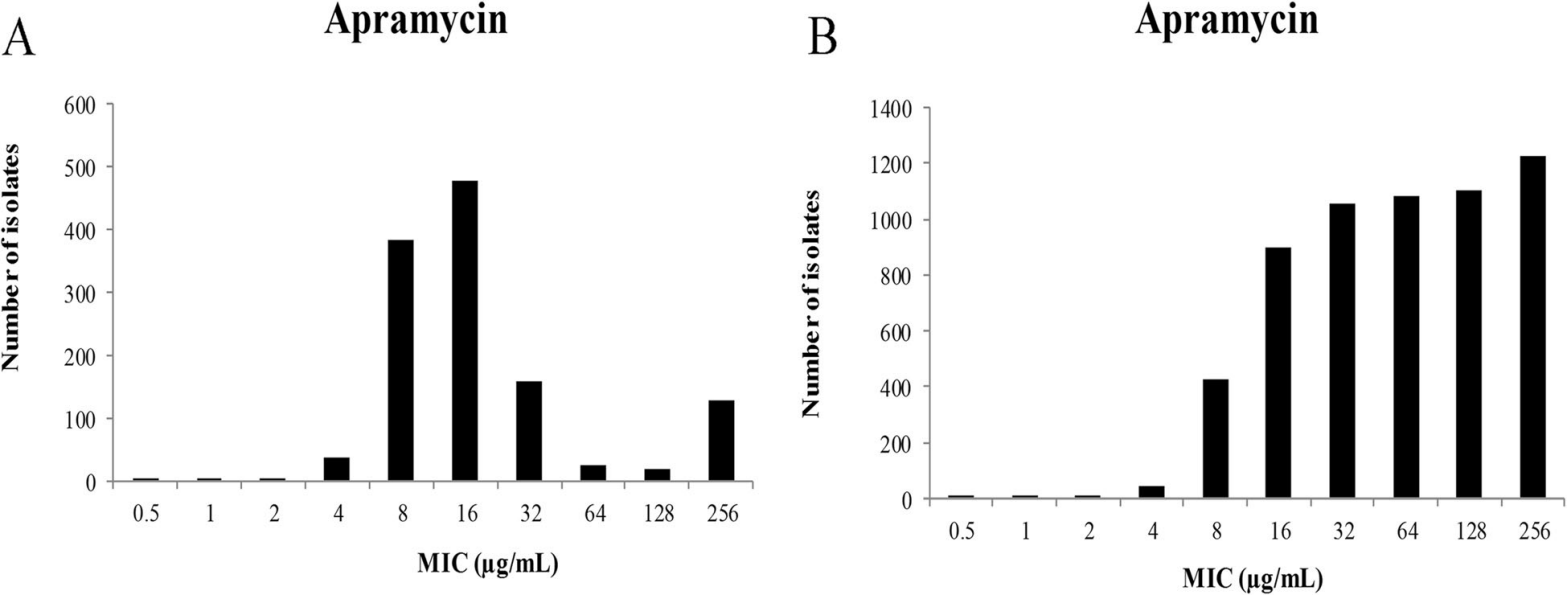
**a**: The original MICs distributions; **b**: cumulative MICs distributions of APR against *E.coli*

The original MICs distributions and MICs cumulative distributions of APR are presented in Fig. 1, MICs for APR against 1230 *E.coli* isolates (858 isolated, 372 donated) were in the range of 0.5 to 256 μg/mL. The percentage of *E. coli* isolates at each MIC (0.5, 1, 2, 4, 8, 16, 32, 64, 128, and 256 μg/mL) were 0.08, 0.08, 0.16, 2.93, 31.14, 38.86, 12.85, 2.03, 1.46, and 10.41%. The MIC_50_ and MIC_90_ were 16 and 64 μg/mL, respectively.

### Establishment of CO_WT_

**Fig. 2:**
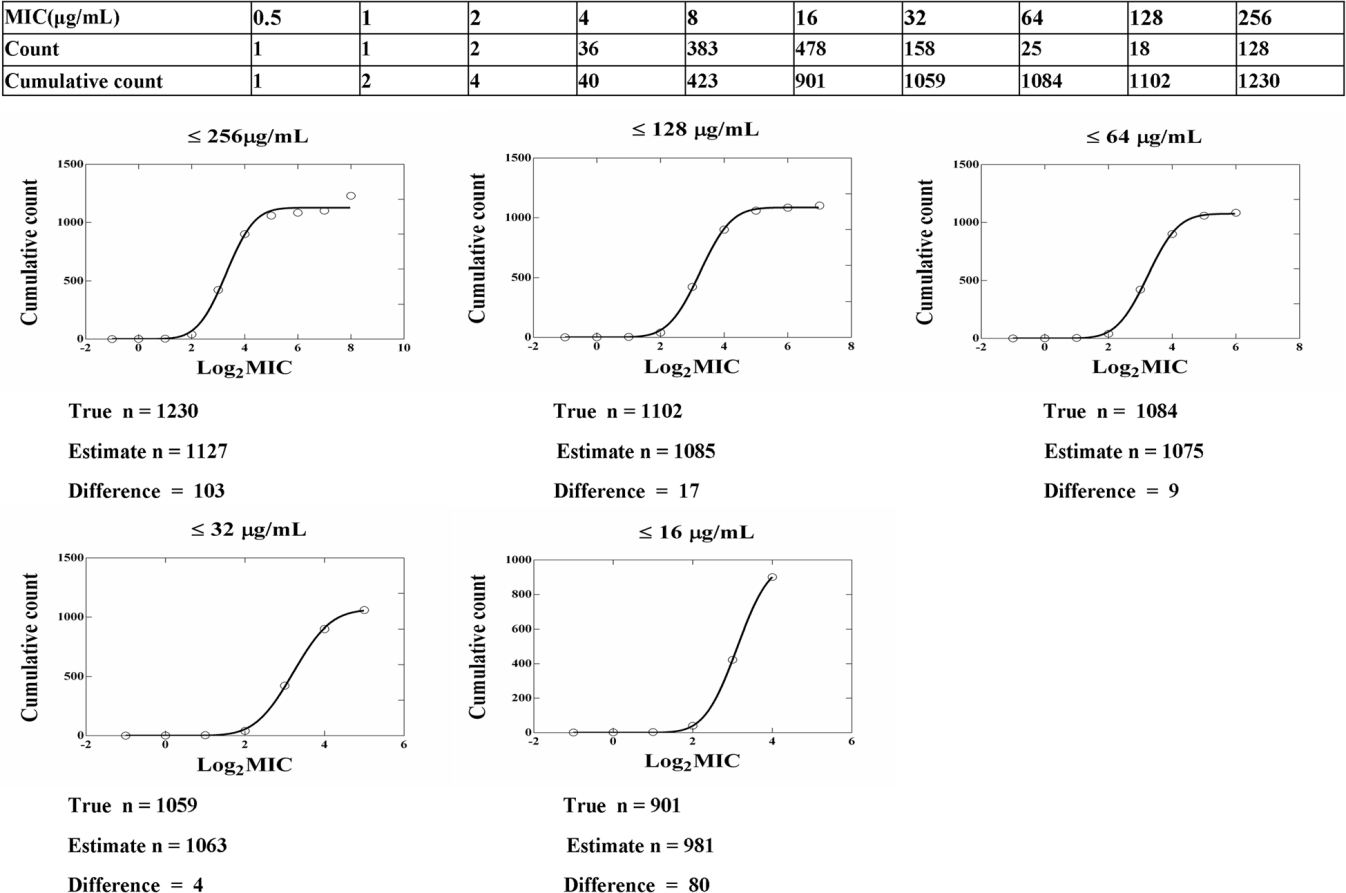
Iterative non-linear regression curve fitting with decreasing subsets. X axis = Log_2_MIC, Y axis = numbers of isolates. Numbers below each graph are the values for the true number of isolates included in the dataset (True n), the non-linear regression estimate (Estimated n) and the difference between these two values of n (Difference). O = observed numbers; solid line = fitted curve

The MIC distributions (1–64 μg/mL) for APR were statistically consistent with a normal distribution (skewness = 0.194 and kurtosis = 0.386). Non-linear regression curve fitting of cumulative log_2_ MIC data was selected as the preferred method for determining the means and standard deviations of MIC distributions owing to the normal (Gaussian) distribution is widely accepted. The process involves fitting an initial subset and generating estimates (in log2) of the strain number, mean and standard deviation in the subset. Repeat this process by reducing the previous subset in each successive column to create the next subset, and repeat the curve fitting until it is clear that there is a subset in which the absolute difference between the true and estimated separation numbers is the smallest. The optimum MIC range from 0.5 to 256 μg/ml was obtained from non-linear regression, the five subsets examined demonstrated that the subset ‘MIC = 32 μg/ mL’ gave the minimum difference (Table 1 and Fig. 2). The probability of an MIC at 32 μg/ml was 99.18%, which encompassed 95% of the WT isolates according to the NORMDIST function in Microsoft Excel (Table 2). As a result, the CO_WT_ was defined as 32 μg/mL.

**Table 1:** Optimum non-linear least squares regression fitting of pooled MICs (μg/mL) for apramycin and *E.coli*

Subset fitted	Number of isolates						Mean MIC (log2)				Standard deviation (log2)			
	TRUE	Est.	Diff.	ASE	Est./ASE	95% CIb	Est.	ASE	Est./ASE	95% CIa	Est.	ASE	Est./ASE	95% CIb
≤256	1230	1127	− 103	25.61	44.00625	1066 to 1188	3.3	0.08125	40.5785	3.105 to 3.489	0.85	0.1107	7.66215	0.5863 to 1.110
≤128	1102	1085	−17	8.104	133.8845	1066 to 1105	3.24	0.02282	141.9369	3.183 to 3.295	0.78	0.03112	25.04177	0.7032 to 0.8555
≤64	1084	1075	−9	8.468	126.9485	1054 to 1097	3.23	0.02011	160.4177	3.174 to 3.277	0.76	0.02713	28.16439	0.6944 to 0.8339
≤32b	1059	1063	4	11.9	89.32773	1030 to 1096	3.21	0.02189	146.6423	3.149 to 3.271	0.75	0.02867	26.03767	0.6669 to 0.8260
≤16	901	981	80	7.849	125.0223	956.3 to 1006	3.11	0.009864	315.3893	3.079 to 3.142	0.64	0.01352	47.20414	0.5952 to 0.6812

Est., non linear regression estimate of value; Diff., estimate of N minus true N; ASE, asymptotic standard error; Est./ASE, estimate divided by asymptotic standard error

a 95% CI of estimate of value

b This subset gave the smallest difference between the estimate and true number of isolates in the subset

**Table 2:** The probability estimation of CO_WT_ with NORMDIST function in microsoft excel

Optimum MIC (μg/mL)	Log_2_ Mean MIC	Mean MIC	Log_2_SD	High cut-off (μg/mL)	Probability of a higher value
≤256	3.21	9.25	0.7465	256	100.00%
≤128	3.21	9.25	0.7465	128	100.00%
≤64	3.21	9.25	0.7465	64	99.99%
≤32^a^	3.21	9.25	0.7465	32	99.18%
≤16	3.21	9.25	0.7465	16	85.50%

^a^the wild type cut-off value

### The prevalence of APR resistance genes

**Fig. 3:**
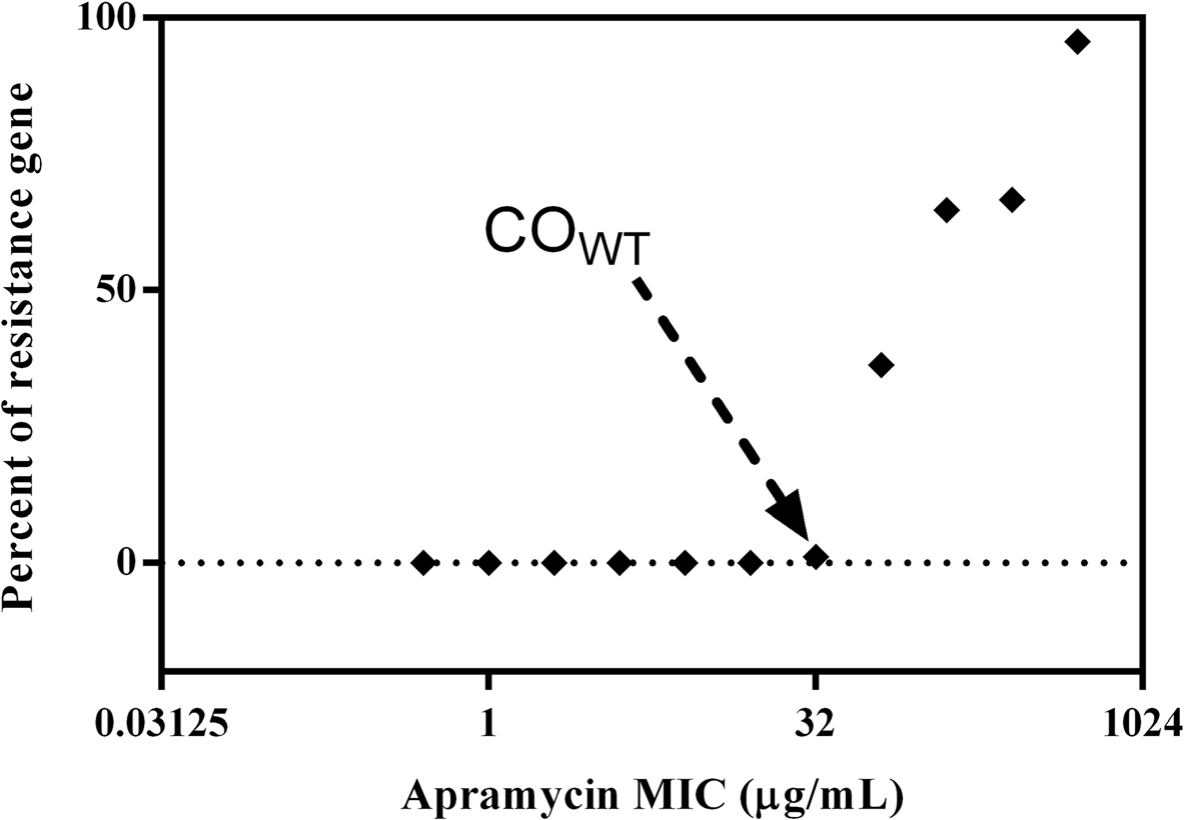
Percentage of *aac(3)-IV* gene in different MIC subsets

A total number of 310 *E.coli* clinical isolates containing different MIC subsets (0.5-256 μg/mL) were conveniently selected for the detection of three resistance genes (*aac(3)-IV*; *npmA*; *apmA*) in *E. coli* by PCR. The prevalence of APR resistance genes presented in Table 3. All the 310 *E.coli* clinical isolates were negative for *npmA* and *apmA* gene by PCR. The only resistance gene in *E.coli* that confer resistance to APR is *aac(3)-IV* in this study. The prevalence of *aac(3)-IV* gene was 91.59% (98/107) in the subset ‘MIC = 256 μg/ mL’; was 64.71% (11/17) in the subset ‘MIC = 128 μg/ mL’; was 36.36% (8/22) in the subset ‘MIC = 64 μg/ mL’; was 1.14% (1/88) in the subset ‘MIC = 32 μg/ mL’ and was 0 in the subset ‘MIC = 0.5-16 μg/ mL’. The percentage of *aac(3)-IV* gene in different MIC subsets is shown in Fig. 3.

**Table 3:** The prevalence of resistance genes that confer resistance to APR in *E. coli*

MIC subset of APR (μg/mL)^a^	Total isolates	Resistance gene (%)		
		Positive no. of *aac(3)-IV*	Positive no. of *npmA*	Positive no. of *apmA*
256	107	98 (91.59%)	0 (0)	0 (0)
128	17	11 (64.71%)	0 (0)	0 (0)
64	22	8 (36.36%)	0 (0)	0 (0)
32	88	1 (1.14%)	0 (0)	0 (0)
16	32	0 (0)	0 (0)	0 (0)
8	20	0 (0)	0 (0)	0 (0)
4	20	0 (0)	0 (0)	0 (0)
2	2	0 (0)	0 (0)	0 (0)
1	1	0 (0)	0 (0)	0 (0)
0.5	1	0 (0)	0 (0)	0 (0)

## Discussion

APR, an aminoglycoside antibiotic, was used in veterinary therapy and animal husbandry in the early 1980’s in several European countries and was approved to use in China since 1999 [4]. However, a recent study demonstrated that APR is a promising drug candidate for the treatment of systemic Gram-negative infections that are resistant to treatment with other aminoglycoside antibiotics by evaluating the in vitro activity of APR against multidrug-, carbapenem- and aminoglycoside resistant Enterobacteriaceae and *Acinetobacter baumannii* in patient from Europe, Asia, Africa and South America [6]. In this study, the results that 171 isolates among the 1230 *E.coli* clinical isolates had MICs ≥64 μg/ mL were similar to the previous study [24]. Resistant *E. coli* are generally isolated from diseased pigs in our study, and *E. coli* from pigs may be an important reservoir for transfer of APR-resistance genes or APR-resistant bacteria to humans [20]. Marshall and Levy, 2011 summarized the evidence from animal on farms to human transfer of resistant bacteria. One is to acquire resistance by direct contact with animals, and the other is the spread of antibiotic resistance through the food chain [25]. The effect of antimicrobial usage on the prevalence of resistant bacteria in animals is significant [26].

Phenotypic resistance is commonly interpreted according to the clinical standards and recommended breakpoints from the European Committee on Antimicrobial Susceptibility Testing (EUCAST) or the CLSI. For aminoglycosides, the MIC breakpoints of amikacin, GEN, netilmicin and tobramycin were established by EUCAST, and the MIC breakpoints of netilmicin, kanamycin, amikacin, tobramycin and GEN were established by CLSI. However, the MIC breakpoint of APR was not established by either EUCAST or CLSI. To set breakpoints required a combination of MIC values, pharmacokinetic/pharmacodynamic relationship and clinical outcome data [21]. However, it is very difficult and expensive to generate this kind of data required for breakpoint determination. The CO_WT_ is a useful tool for the interpretation of antimicrobial susceptibility testing results conducted in laboratories [21]. In this study, the CO_WT_ was defined as ≤32 μg/mL by using a statistical method recommended by CLSI and was similar with that the epidemiological cut-off value (ECOFF) routinely used for APR was >16 μg/ mL by the Laboratory of Swine diseases, Kjellerup, Denmark and by the Danish Veterinary Institute, Frederiksberg, Denmark [27]. Different use of apramycin in pigs and chickens results in different susceptibility of clinical *E. coli* strains to apramycin. Tian et al. 2019 reported that “from 2016 to 2018, a total of 1412 *E. coli* from chickens were identified in 10 Chinese provinces. MIC _50_ and MIC _90_ for apramycin against *E. coli* (0.5~256 μg/mL) were 8 and 16 μg/mL, respectively.” [28]. They conclude that the ECV (CO_WT_) for APR in *E. coli* is 16 μg/mL. The percentage of *E. coli* isolates at each MIC (0.5 to 256 μg/mL) is very different between Tian et al. 2019 and this study. Therefore, we think that the CO_WT_ (ECV) different form Tian et al. 2019 is reasonable.

To date, it has been determined that two resistance genes in *E. coli* (*aac(3)-IV*, *npmA*) confer resistance to APR [13, 14]. The gene *aac(3)-IV* is the only identified gene causing enzymatic cross-resistance between APR and GEN [29]. GEN is a critically important drug and is generally combinated with beta-lactam as the first choice antimicrobial for severe human infections [19]. In this study, the high prevalence of *aac(3)-IV* gene was observed in the resistant *E.coli* isolates, which was consistant with other previous studies [4, 19, 30–32]. The *npmA* gene, confers high resistance to many aminoglycoside types upon the host *E. coli*, was originally found in an *E.coli* strain isolated in 2003 from the urine of an inpatient in a general hospital in Japan [14] and did not appear in the scientific literature until August 2017 from China [33]. The *npmA* gene was not detected in any samples in this study, which consistant with other previous study [34]. The *apmA* gene was at first detected in bovine methicillin-resistant *Staphylococcus aureus* (MRSA) of sequence type 398 in 2011 [15] and was not found in any isolates in our study. Due to only the *aac(3)-IV* gene was found in all APR resistant isolates tested, suggesting that it is the predominant gene responsible for this resistance pattern in the pigs. The risk of transfer of APR/GEN cross-resistant resistant gene *aac(3)-IV* in *E.coli* from animals to humans is of great concern.

## Conclusion

Given the lack of interpretion criteria of APR susceptibility testing, the CO_WT_ (≤ 32 μg/mL) for APR against *E.coli* was established by using a statistical method recommended by CLSI in this study. The prevelance of APR resistance gene *aac(3)-IV* mainly concentrated in these MIC subsets “MIC ≥ 64 μg/ mL”, which indicates that the CO_WT_ established in our study is reliable. The CO_WT_ offers guidance for APR susceptibility testing of *E.coli* isolated from animals.

## Methods

### Isolates

The rectal swabs collected on each visit from the target animals were pooled and tested as one analytical sample. A total of 1230 *E.coli* isolates were used in the study, which including 858 isolates identified from rectal swabs of pigs in different province in China by using the biochemical identification and PCR method according to ‘Bergey’s Manual of Determinative Bacteriology’ [35]: Heilongjiang (*n* = 293), Jilin (*n* = 151), Liaoning (*n* = 238), Henan (*n* = 97), Shandong (*n* = 30), Hubei (*n* = 20), and Yunnan (n = 29) from June 2014 to April 2017, and 372 *E.coli* strains were respectively donated by National Key Laboratory of Veterinary Biotechnology, Harbin Veterinary Research Institute, Chinese Academy of Agricultural Sciences (*n* = 108), Husbandry and Veterinary College, Jilin University (*n* = 112), and College of Animal Husbandry and Veterinary Science, Henan Agricultural University (*n* = 152). All of the bacterial isolates were confirmed by polymerase chain reaction (PCR) [36].

### Chemicals and reagents

Pure powder of APR was purchased from Zhejiang Hisun Pharmaceutical Co., Ltd., Taizhou, China. MacConkey medium, eosin-methylene blue medium, Mueller-Hinton (MH) broth, and MH agar were supplied form Qingdao Hope Bio-Technology Co., Ltd., Qingdao, China. Premix Taq™ Version 2.0 plus dye and DL1000 DNA Marker were obtained from Takara Biotechnology Co., Dalian, China. All primers used in the study were synthesized by the Sangon Biotech Co., Ltd., Shanghai, China.

### Antibacterial susceptibility testing

Broth microdilution testing was performed according to the CLSI document M07-A9 [37] at the following laboratories: Department of Microbiology, Department of Pharmacology and Toxicology, and Pharmacy Department in Northeast Agricultural University, Harbin, China. APR stock solution of 5120 μg/mL was prepared. Working solutions in plates were prepared by two-fold serial dilutions in MH broth. Finally, each well of 96 well plates contains approximately 5 × 10^5^ CFU/mL *E.coli* and APR concentrations ranged from 0.5 to 256 μg/mL. Plates were placed in a constant temperature incubator at 37 °C for 20 h. Quality control (QC) isolate *E.coli* ATCC 25922 (purchased from the NATIONAL CENTER FOR MEDICAL CULTURE COLLECTIONS, Beijing, China) was used on each day of testing as recommended by CLSI [37]. Only those results, for which the QC MICs were within the established reference range (4-8 μg/mL), were used in the study [38]. All MICs determinations were performed in triplicate.

### Data analysis

The definitions of the subsets, lognormal distribution, skewness, kurtosis, and CO_WT_ are presented in Table 4. The MICs were transformed into log_2_ values in order to analyze the MIC distributions. The kurtosis and skewness of each MIC distribution were tested. To confirm the presence of more than one MIC distribution, frequency distributions of MIC data were analyzed by nonlinear least squares regression analysis based on the following Cumulative Gaussian Counts equation: Z = ((X-Mean))/SD, Y=N*zdist(z) according to the previous study [41], in which the Mean is the average of the original distribution, from which the frequency distribution was created; SD is the standard deviation of the original distribution (calculated by Graphpad prism 6.0 software, San Diego, CA). Three parameters, the total number (N) in the presumed unimodal distribution, the mean, and SD (both log_2_) were estimated. N was estimated rather than taken as a constant in the regression, because of the desire to fit the data to the distribution without assuming that N truly contained only wild-type isolates [22]. The NORMINV and NORDIST functions in Microsoft Excel were used to set the WT distribution cutoffs which were used to determine the MIC that encompass at least 95% of that distribution [22, 42].

**Table 4:** Definitions of the terminology used in this study

Terminology	Description	Reference
Subsets	Subsets of data extracted from datasets	[22]
Lognormal Distribution	A frequency (probability) distribution where the data are distributed in a Gaussian (normal) manner after the data points have been converted to logarithms.	[22]
Skewness	Lack of symmetry in a frequency distribution.	[22]
Kurtosis	Excessive peaking or flattening of a frequency distribution when compared with the normal distribution.	[22]
CO_WT_	CO_WT_ also known as the epidemiological cutoff (ECV), defined as the highest susceptibility endpoint of the wild-type (WT) population MIC, has been shown to detect the emergence of in vitro resistance or to separate WT isolates (without known mechanisms of resistance) from non-WT isolates (with mechanisms of resistance and reduced susceptibilities to the antibacterial agent being evaluated). CO_WT_ are calculated by taking into account the MIC distribution, the modal MIC of each distribution, and the inherent variability of the test (usually within one doubling dilution) and should encompass ≥95% of isolates.	[22, 39, 40]

### Molecular characterisation of mechanisms of resistance to APR

A total number of 310 *E.coli* strains from different MIC subsets (0.5–256 μg/mL) were conveniently selected for the detection of resistance genes in *E. coli* that confer resistance to APR by PCR. The primers used in this study are presented in Table 5. Genomic DNA was extracted using a TIANamp Bacteria DNA Kit (TIANGEN BIOTECH (BEIJING) CO., LTD.) according to the manufacturer’s instructions. Then, 2 μL (400 ng/μL) was added to a reaction mixture containing 25 μL Premix TaqTM Version 2.0 plus dye, 13 μL sterile ddH_2_O, 5 μL 10 μM primer F and 5 μL 10 μM primer R. Amplification conditions were 94 °C for 5 min, followed by 30 cycles of 94 °C for 30 s, 55 °C for 30 s (52 °C for *apmA*) and 72 °C for 1 min, and a final elongation at 72 °C for 10 min. PCR products were analysed on 1.5% (w/v) agarose gelsstained with ethidium bromide. The amplified products were sequenced by the Sangon Biotech Co., Ltd., Shanghai, China. *E. coli* ATCC 25922 strains was used as negative controls.

**Table 5:** The primers used in the detection of APR resistance genes and expected amplicon sizes

Gene	DNA sequence (5′–3′)		Product (bp)	Reference
*aac(3)-IV*	*aac(3)-IV* F *aac(3)-IV* R	TCGGTCAGCTTCTCAACCTT GATGATCTGCTCTGCCTGTG	314	[43]
*npmA*	*npmA* F *npmA* R	CTCAAAGGAACAAAGACGG GAAACATGGCCAGAAACTC	641	[43]
*apmA*	*apmA* F *apmA* R	CGTTTGCTTCGTGCATTAAA TTGACACGAAGGAGGGTTTC	656	[15]

## Data Availability

The datasets used and analyzed in this study are available from the corresponding author on reasonable request.

## Abbreviations

*E.coli*: *Escherichia coli*
ETEC: Enterotoxigenic *E.coli*
APR: Apramycin
GEN: Gentamicin
CO_WT_: Wild-type cutoff values
CLSI: Clinical and Laboratory Standards Institute
EUCAST: European Committee on Antimicrobial Susceptibility Testing

## References

[1] Dubreuil JD (2012). The whole shebang: the gastrointestinal tract, *Escherichia coli* enterotoxins and secretion. Curr Issues Mol Biol.

[2] Kotloff KL, Nataro JP, Blackwelder WC, Nasrin D, Farag TH, Panchalingam S, Wu Y, Sow SO, Sur D, Breiman RF (2013). Burden and aetiology of diarrhoeal disease in infants and young children in developing countries (the global enteric multicenter study, GEMS): a prospective, case-control study. Lancet.

[3] Fairbrother JM, Nadeau E, Gyles CL (2005). Escherichia coli in postweaning diarrhea in pigs: an update on bacterial types, pathogenesis, and prevention strategies. Anim Health Res Rev.

[4] Zhang XY, Ding LJ, Fan MZ (2009). Resistance patterns and detection of aac(3)-IV gene in apramycin-resistant Escherichia coli isolated from farm animals and farm workers in northeastern of China. Res Vet Sci.

[5] Riedel S, Vijayakumar D, Berg G, Kang AD, Smith KP, Kirby JE (2019). Evaluation of apramycin against spectinomycin-resistant and -susceptible strains of Neisseria gonorrhoeae. J Antimicrob Chemother.

[6] Juhas M, Widlake E, Teo J, Huseby DL, Tyrrell JM, Polikanov YS, Ercan O, Petersson A, Cao S, Aboklaish AF (2019). In vitro activity of apramycin against multidrug-, carbapenem- and aminoglycoside-resistant Enterobacteriaceae and Acinetobacter baumannii. J Antimicrob Chemother.

[7] Truelson KA, Brennan-Krohn T, Smith KP, Kirby JE (2018). Evaluation of apramycin activity against methicillin-resistant, methicillin-sensitive, and vancomycin-intermediate Staphylococcus aureus clinical isolates. Diagn Microbiol Infect Dis.

[8] Moore JE, Koulianos G, Hardy M, Misawa N, Millar BC (2018). Antimycobacterial activity of veterinary antibiotics (Apramycin and Framycetin) against Mycobacterium abscessus: implication for patients with cystic fibrosis. Int J Mycobacteriol.

[9] Kang AD, Smith KP, Berg AH, Truelson KA, Eliopoulos GM, McCoy C, Kirby JE. Efficacy of Apramycin against multidrug-resistant Acinetobacter baumannii in the murine neutropenic thigh model. Antimicrob Agents Chemother. 2018;62(4). 10.1128/AAC.02585-17 PMC5913965 29339396

[10] Kang AD, Smith KP, Eliopoulos GM, Berg AH, McCoy C, Kirby JE (2017). Invitro Apramycin activity against multidrug-resistant Acinetobacter baumannii and Pseudomonas aeruginosa. Diagn Microbiol Infect Dis.

[11] Hu Y, Liu L, Zhang X, Feng Y, Zong Z (2017). In vitro activity of neomycin, streptomycin, Paromomycin and Apramycin against Carbapenem-resistant Enterobacteriaceae clinical strains. Front Microbiol.

[12] Wray C, Hedges RW, Shannon KP, Bradley DE (1986). Apramycin and gentamicin resistance in Escherichia coli and salmonellas isolated from farm animals. J Hyg.

[13] Davies J, O'Connor S (1978). Enzymatic modification of aminoglycoside antibiotics: 3-N-acetyltransferase with broad specificity that determines resistance to the novel aminoglycoside apramycin. Antimicrob Agents Chemother.

[14] Wachino J, Shibayama K, Kurokawa H, Kimura K, Yamane K, Suzuki S, Shibata N, Ike Y, Arakawa Y (2007). Novel plasmid-mediated 16S rRNA m1A1408 methyltransferase, NpmA, found in a clinically isolated Escherichia coli strain resistant to structurally diverse aminoglycosides. Antimicrob Agents Chemother.

[15] Fessler AT, Kadlec K, Schwarz S (2011). Novel apramycin resistance gene apmA in bovine and porcine methicillin-resistant Staphylococcus aureus ST398 isolates. Antimicrob Agents Chemother.

[16] Johnson AP, Malde M, Woodford N, Cunney RJ, Smyth EG (1995). Urinary isolates of apramycin-resistant Escherichia coli and Klebsiella pneumoniae from Dublin. Epidemiol Infect.

[17] Yates CM, Pearce MC, Woolhouse ME, Amyes SG (2004). High frequency transfer and horizontal spread of apramycin resistance in calf faecal Escherichia coli. J Antimicrob Chemother.

[18] Johnson AP, Burns L, Woodford N, Threlfall EJ, Naidoo J, Cooke EM, George RC (1994). Gentamicin resistance in clinical isolates of Escherichia coli encoded by genes of veterinary origin. J Med Microbiol.

[19] Jensen VF, Jakobsen L, Emborg HD, Seyfarth AM, Hammerum AM (2006). Correlation between apramycin and gentamicin use in pigs and an increasing reservoir of gentamicin-resistant Escherichia coli. J Antimicrob Chemother.

[20] Johnson JR, Kuskowski MA, Smith K, O'Bryan TT, Tatini S (2005). Antimicrobial-resistant and extraintestinal pathogenic Escherichia coli in retail foods. J Infect Dis.

[21] Lockhart SR, Ghannoum MA, Alexander BD (2017). Establishment and use of epidemiological cutoff values for molds and yeasts by use of the clinical and laboratory standards institute M57 standard. J Clin Microbiol.

[22] Turnidge J, Kahlmeter G, Kronvall G (2006). Statistical characterisation of bacterial wild-type MIC value distributions and the determination of epidemiological cut-off values. Clin Microbiol Infect.

[23] Kronvall G (2010). Normalized resistance interpretation as a tool for establishing epidemiological MIC susceptibility breakpoints. J Clin Microbiol.

[24] Smith M, Do TN, Gibson JS, Jordan D, Cobbold RN, Trott DJ (2014). Comparison of antimicrobial resistance phenotypes and genotypes in enterotoxigenic Escherichia coli isolated from Australian and Vietnamese pigs. J Glob Antimicrob Resist.

[25] Marshall BM, Levy SB (2011). Food animals and antimicrobials: impacts on human health. Clin Microbiol Rev.

[26] Berendonk TU, Manaia CM, Merlin C, Fatta-Kassinos D, Cytryn E, Walsh F, Burgmann H, Sorum H, Norstrom M, Pons MN (2015). Tackling antibiotic resistance: the environmental framework. Nat Rev Microbiol.

[27] Agersø Y, Hald T, Helwigh B, Høg BB, Jensen LB, Jensen VF, Korsgaard H, Larsen LS, Seyfarth AM, Struve T (2012). DANMAP 2011 - use of antimicrobial agents and occurrence of antimicrobial resistance in bacteria from food animals, food and humans in Denmark. J Veg Sci.

[28] Erjie T, Ishfaq M, Wanjun H, Zhiyong W, Rui L, Xiaoxiao L, Chunli C, Jichang L (2019). Tentative epidemiologic cut-off value and resistant characteristic detection of apramycin against Escherichia coli from chickens. FEMS Microbiol Lett.

[29] Chaslus-Dancla E, Pohl P, Meurisse M, Marin M, Lafont JP (1991). High genetic homology between plasmids of human and animal origins conferring resistance to the aminoglycosides gentamicin and apramycin. Antimicrob Agents Chemother.

[30] Mathew AG, Arnett DB, Cullen P, Ebner PD (2003). Characterization of resistance patterns and detection of apramycin resistance genes in Escherichia coli isolated from swine exposed to various environmental conditions. Int J Food Microbiol.

[31] Mathew AG, Garner KN, Ebner PD, Saxton AM, Clift RE, Liamthong S (2005). Effects of antibiotic use in sows on resistance of E. coli and salmonella enterica typhimurium in their offspring. Foodborne Pathog Dis.

[32] Choi MJ, Lim SK, Nam HM, Kim AR, Jung SC, Kim MN (2011). Apramycin and gentamicin resistances in indicator and clinical Escherichia coli isolates from farm animals in Korea. Foodborne Pathog Dis.

[33] Zhao Z, Lan F, Liu M, Chen W, Huang L, Lin Q, Li B (2017). Evaluation of automated systems for aminoglycosides and fluoroquinolones susceptibility testing for Carbapenem-resistant Enterobacteriaceae. Antimicrob Resist Infect Control.

[34] Zhang A, Li Y, Guan Z, Tuo H, Liu D, Yang Y, Xu C, Lei C, Wang H (2018). Characterization of resistance patterns and detection of Apramycin resistance genes in Escherichia coli isolated from chicken feces and houseflies after Apramycin administration. Front Microbiol.

[35] Buchanan R, Gibbons N. Bergey's Manual of Systematic Bacteriology. William and Wilkens; 1984.

[36] Seurinck S, Verstraete W, Siciliano SD (2003). Use of 16S-23S rRNA intergenic spacer region PCR and repetitive extragenic palindromic PCR analyses of Escherichia coli isolates to identify nonpoint fecal sources. Appl Environ Microbiol.

[37] CLSI (2012). CLSI document M07-A9.

[38] Odland BA, Erwin ME, Jones RN (2000). Quality control guidelines for disk diffusion and broth microdilution antimicrobial susceptibility tests with seven drugs for veterinary applications. J Clin Microbiol.

[39] Kahlmeter G, Brown DF, Goldstein FW, MacGowan AP, Mouton JW, Osterlund A, Rodloff A, Steinbakk M, Urbaskova P, Vatopoulos A (2003). European harmonization of MIC breakpoints for antimicrobial susceptibility testing of bacteria. J Antimicrob Chemother.

[40] Turnidge J, Paterson DL (2007). Setting and revising antibacterial susceptibility breakpoints. Clin Microbiol Rev.

[41] Yang Y, Zhang Y, Li J, Cheng P, Xiao T, Muhammad I, Yu H, Liu R, Zhang X (2019). Susceptibility breakpoint for Danofloxacin against swine Escherichia coli. BMC Vet Res.

[42] Espinel-Ingroff A, Colombo AL, Cordoba S, Dufresne PJ, Fuller J, Ghannoum M, Gonzalez GM, Guarro J, Kidd SE, Meis JF (2016). International evaluation of MIC distributions and epidemiological cutoff value (ECV) definitions for Fusarium species identified by molecular methods for the CLSI broth microdilution method. Antimicrob Agents Chemother.

[43] Fernandez-Martinez M, Miro E, Ortega A, Bou G, Gonzalez-Lopez JJ, Oliver A, Pascual A, Cercenado E, Oteo J, Martinez-Martinez L (2015). Molecular identification of aminoglycoside-modifying enzymes in clinical isolates of Escherichia coli resistant to amoxicillin/clavulanic acid isolated in Spain. Int J Antimicrob Agents.

